# Linking Cellular Mechanisms to Behavior: Entorhinal Persistent Spiking and Membrane Potential Oscillations May Underlie Path Integration, Grid Cell Firing, and Episodic Memory

**DOI:** 10.1155/2008/658323

**Published:** 2008-07-21

**Authors:** Michael E. Hasselmo, Mark P. Brandon

**Affiliations:** Center for Memory and Brain, Department of Psychology and Program in Neuroscience, Boston University, 2 Cummington Sreet, Boston, MA 02215, USA

## Abstract

The entorhinal cortex plays an important role in spatial memory and episodic memory functions. These functions may result from cellular mechanisms for integration of the afferent input to entorhinal cortex. This article reviews physiological data on persistent spiking and membrane potential oscillations in entorhinal cortex then presents models showing how both these cellular mechanisms could contribute to properties observed during unit recording, including grid cell firing, and how they could underlie behavioural functions including path integration. The interaction of oscillations and persistent firing could contribute to encoding and retrieval of trajectories through space and time as a mechanism relevant to episodic memory.

## 1. INTRODUCTION

The entorhinal cortex plays an important role in memory function. In the rat, entorhinal cortex lesions impair performance in both spatial memory tasks [1] as well as in odor memory tasks [2]. In monkeys, memory function in delayed match to sample tasks is impaired by lesions of entorhinal cortex [3] and adjacent parahippocampal structures [4]. This article describes cellular and circuit mechanisms in the entorhinal cortex that could underlie its role in spatial and episodic memory functions. Computational modeling links data across multiple levels of function, including: (a) properties of single cell physiology, including persistent spiking and membrane potential oscillations, (b) properties of unit recording, including grid cells, place cells, and head direction cells, and (c) the role of entorhinal cortex in spatial path integration and episodic memory function. This article will review physiological data and modeling across these different levels.

## 2. PHYSIOLOGICAL DATA

Recordings from neurons in slice preparations of entorhinal cortex demonstrate important cellular properties including (i) persistent spiking and (ii) membrane potential oscillations. These cellular properties could contribute to properties described in unit recordings from entorhinal cortex in awake, behaving rats.

### 2.1. Persistent spiking

**Figure 1:**
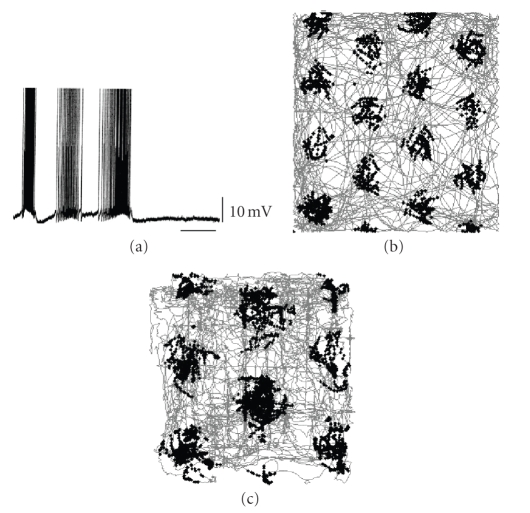
(a) Example of persistent firing in layer II pyramidal cell showing alternating cycles of spiking and nonspiking in data from Klink and Alonso [5]. (b) Simulation of grid cell firing dependent upon cyclical persistent spiking gated by random movement in a two-meter square environment. Spiking shown as black dots on trajectory in gray. (c) Simulation from same model using rat trajectory from experimental data in an 85 cm square environment.

**Figure 2:**
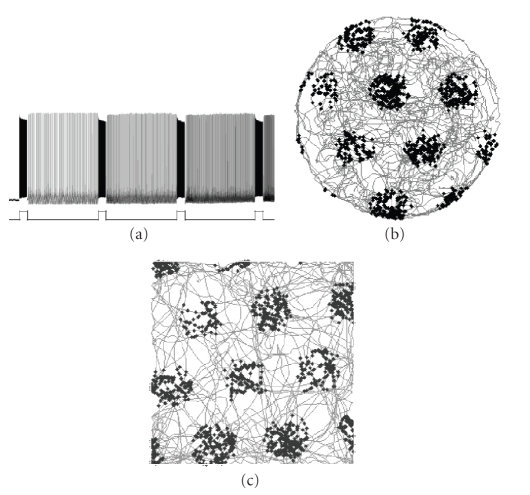
(a) Example of graded persistent firing in a layer V pyramidal cell from Egorov et al. [8]. (b) Simulation of grid cell firing based on persistent firing in cells from deep layers of medial entorhinal cortex. The spiking activity shown as black dots arises from convergent input from three neurons with the same baseline persistent firing frequency, with phase of input neurons influenced by input from different speed modulated head direction cells during movement (trajectory shown in gray). (c) Simulation of grid cell firing based on membrane potential oscillations in dorsal layer II stellate cells in medial entorhinal cortex.

In slices, pyramidal neurons in different layers of entorhinal cortex demonstrate the capacity to display persistent spiking activity after a depolarizing current injection or a period of repetitive synaptic input [5–8]. Pyramidal neurons in layer II of medial entorhinal cortex show persistent spiking that tends to turn on and off over periods of many seconds [5]. This cyclical persistent spiking is shown in Figure 1(a). As described below, this could underlie the spatial periodicity of grid cells. Pyramidal neurons in deep layers of entorhinal cortex can maintain spiking at different graded frequencies for many minutes [8] as shown in Figure 2(a). The persistent spiking appears to due to muscarinic or metabotropic glutamate activation of a calcium-sensitive nonspecific cation current [7, 9, 10]. This graded persistent firing could allow these neurons to integrate synaptic input over extended periods. Persistent firing has also been shown in layer III of lateral entorhinal cortex [6].

### 2.2. Membrane potential oscillations

Entorhinal layer II stellate cells show prominent subthreshold membrane potential oscillations when depolarized near firing threshold [11, 12]. These are small oscillations of a few millivolts in amplitude that can influence the timing of action potentials [13] and can contribute to network oscillations [14, 15]. The frequency of membrane potential oscillations differs systematically along the dorsal to ventral axis of the medial entorhinal cortex [16]. A model presented below discusses how the membrane potential oscillation properties could underlie differences in grid cell firing properties along the dorsal to ventral axis [16–19]. The oscillations appear to be due to a hyperpolarization activated cation current or h-current [20], that differs in time constant along the dorsal to ventral axis [21]. Depolarizing input increases the frequency of these oscillations such that the phase of the oscillation integrates the depolarizing input over time. Membrane potential oscillations do not usually appear in layer II or layer III pyramidal cells [12], but are observed in layer V pyramidal cells, where they may be caused by M-current [22]. Membrane potential oscillations do not appear in neurons of the lateral entorhinal cortex [23].

### 2.3. Unit recording data

Recordings of neural activity in awake behaving rats provide important clues to the functional role of entorhinal cortex. In particular, many cells in medial entorhinal cortex fire as “grid cells.” A single grid cell responds as a rat forages in a hexagonal array of different locations in an open-field environment [24, 25]. Examples of the pattern of firing in modeled grid cells are shown in Figures 1 and 2. Grid cells differ in spatial periodicity along the dorsal to ventral axis of medial entorhinal cortex, with larger spacing between larger fields in more ventral regions [24, 25].

Grid cells appear in all layers of entorhinal cortex, but in layers V and VI of entorhinal cortex the grid cells often only respond when the rat is facing in a particular direction [25]. This resembles head direction cells in areas such as the postsubiculum (dorsal presubiculum), which respond at all locations in the environment but only when the rat faces a particular direction [26–30]. The conjunctive grid-by-direction cells resemble the theta-modulated place-by-direction cells observed in the post- and parasubiculum, which respond only when the rat faces a preferred direction while occupying a single location [31].

## 3. PATH INTEGRATION

**Figure 3:**
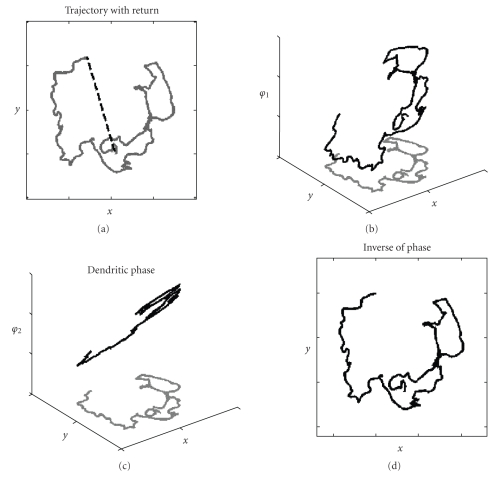
Coding of location by phase. (a) Actual trajectory run by the rat is shown in gray. If phase is reset at start location, the inverse transform of phase at any position yields a return vector with angle and distance leading back to start (shown with black dashed line). (b) Plot of membrane potential oscillation phase *φ* in a single dendrite of a simulated grid cell, showing linear change in phase with one dimension of location (dendrite receives input from head direction cell with angle preference zero). (c) Phase of another dendrite receiving input from head direction with angle preference 120. (d) Performing the inverse transform of oscillation phases at each point in time effectively reconstructs the full trajectory.

The cellular mechanisms described above may contribute to the function of path integration. Path integration involves an animal using its self-motion cues to maintain an accurate representation of the angle and distance from its start position, even during performance of a complex trajectory through the environment [32–35]. Many species demonstrate the behavioral capacity to remember the distance as well as the angle of return to the starting location (here represented in Cartesian coordinates by a two component return vector). As shown here, persistent firing provides a single neuron mechanism to integrate the distance and angle of trajectory segments to compute the overall distance and angle from start location to an end or goal location. Here, the vector from start to goal is called the goal vector (and the negative of the goal vector is called the return vector). An example of a return vector is shown as a dashed line in Figure 3(a).

In general, the location of an agent can be determined by computing the integral of the velocity vector. In Cartesian coordinates, the velocity vector is $\vec{v}\;=\;[dx/dt,\;dy/dt]$. Integration of the velocity vector over a period of time *T* after starting at location ${\vec{x}}_{0}$ yields the location vector $\vec{x}=\;[x,\;y]$ at time *T*:

(1) $$\vec{x}(T)\;=\;{\vec{x}}_{0}\;+\;\int_{0}^{T}\vec{v}(t)dt$$.

For example, if the velocity of an animal is 10 cm/sec in the *x* direction and 5 cm/sec in the *y* direction, integration over 5 seconds yields a final location of [*x*, *y*] = [50 cm, 25 cm] relative to the start location. Note that this integral corresponds to the goal vector $\overset{⇀}{g}(T)\;=\;\overset{⇀}{x}(T)-{\overset{⇀}{x}}_{0}$, which is the negative of the return vector.

## 4. INTEGRATION BY PERSISTENT FIRING CAN CODE LOCATION

Integration of the goal vector or return vector could be provided by the mechanism of graded persistent firing in deep layer entorhinal neurons [7, 8]. These neurons could integrate a velocity vector coded by neurons responsive to the head direction of the rat and to the speed of the rat. Head direction cells have been shown in deep entorhinal cortex [25] and in the postsubiculum, which provides direct input to the entorhinal cortex [26, 27]. Head direction cells respond selectively when the rat is heading in a specific allocentric direction. Some neurons show sensitivity to speed (translational motion) in the postsubiculum [27] as well as in the hippocampus [36] and in the medial mammillary nucleus which receives output from the postsubiculum and medial entorhinal cortex [37]. Here, the activity of a population of head direction cells modulated by speed is represented by multiplying the velocity vector of the rat with a head direction matrix *H*. The head direction matrix consists of rows with unit vectors representing the preference angles of individual speed modulated head direction cells, that transforms the velocity vector into a head direction vector $\overset{⇀}{h}\;=\;H\overset{⇀}{v}$. For example, a matrix representing two head direction cells with preference angles *θ*_1_ and *θ*_2_ has two rows: $H\;=\;\left[\begin{array}{cc} \cos\;\theta_{1} & \sin\,\;\theta_{1}\\ \cos\,\;\theta_{2} & \sin\,\;\theta_{2} \end{array}\right]$. For a cell with a preference angle of 0, the activity of the head direction cell would be cos(0)∗*d* *x*/*d* *t* + sin(0)*d* *y*/*d* *t* = *d* *x*/*d* *t*. This framework has the advantage that it allows for a simple inverse transform turning head direction space back into Cartesian coordinates. The inverse [38] of the matrix *H* is

(2) $$H^{-1}\;=\;\left[\begin{array}{cc} \sin\theta_{2} & -\sin\theta_{1}\\ -\cos\theta_{2} & \cos\theta_{1} \end{array}\right]/(\cos\theta_{1}\sin\theta_{2}-\sin\theta_{1}\cos\,\;\theta_{2}).$$

Note that this framework assumes that the response of head direction cells is like a cosine function, whereas head direction cells usually only show positive activity and have narrower, triangular response functions with no activity outside this range. Head direction input corresponding to a cosine function of actual input could be provided by summed input converging from a population of head direction cells with different magnitudes of tuning for values at different angles from the preferred direction. Note also that this representation combines the properties of different neurons responding to translational velocity or to head direction in the postsubiculum [27] and other regions [36, 37]. If head direction is not computed based on velocity, then it could be integrated from angular velocity as a distinct element of the state vector or could be computed based on angle to a different reference point.

Using this mathematical representation of head direction input, the firing rate of a set of graded persistent firing cells could integrate the input from a set of head direction cells to yield a firing rate as follows:

(3) $$\overset{⇀}{a}(T)\;=\;\beta \int_{t=0}^{T}H(\overset{⇀}{v}(t)dt)\;=\;\beta H(\overset{⇀}{x}(T)\;-\;\overset{⇀}{x}(0)),$$

where vector $\vec{a}(T)$ represents the firing rate of a population of graded persistent firing cells at time *T*. For example, imagine two cells *a*_1_ and *a*_2_ with capacity for graded persistent spiking that receive input from two head direction cells with preference angles of 0 degrees and 60 degrees. Imagine that the rat moves at 10 cm/sec in the *x* direction for 4 seconds, and the scaling factor *β* is 0.25 Hz/cm. Moving in the *x* direction is equivalent to moving at 0 degrees, which would result in the head direction cells having the activity *h*_1_ = 1 and *h*_2_ = 0.5. The computation in (3) would then result in the frequency of the graded persistent firing cells as follows: $a\;=\;\beta \left[\begin{array}{cc} \cos\,\;\theta_{1} & \sin\,\;\theta_{1}\\ \cos\,\;\theta_{2} & \sin\,\;\theta_{2} \end{array}\right]\left[\begin{array}{c} x\\ y \end{array}\right]\;=\;0.25\left[\begin{array}{cc} 1 & 0\\ 0.5 & 0.87 \end{array}\right]\left[\begin{array}{c} 40\\ 0 \end{array}\right]\;=\;\left[\begin{array}{c} 10\\ 5 \end{array}\right]$. Thus, the graded persistent firing cells would increase their activity to *a*_1_ = 10 Hz and *a*_2_ = 5 Hz. Mathematically, the inverse transform of this firing rate vector computes the location vector $\vec{x}(T)\;=\;H^{-1}\vec{a}(T)/\beta$ in Cartesian coordinates (see Figure 3(a)). However, the difference in neural activity could guide behavior without use of the inverse transform. This could involve forming associations between the start location and this pattern of graded firing, and then forming associations between this pattern of graded firing and the associated head direction signal. At the start location, the pattern of graded firing could be activated, and this could retrieve the associated head direction signal. The animal could change directions until its actual head direction matched this retrieved head direction. This could give the animal the correct angle to the goal.

The same mechanism computes both the goal vector and its negative, the return vector. The return vector allows a rat to return to the starting location from any arbitrary location in the environment. In contrast, the goal vector can be used to store the distance and angle to important locations in the environment. For example, if a rat is started in one location in an open field, and wanders until it finds food in another location, the integrated activity vector at that point is the goal vector—it provides a simple description of the angle and distance from the start to the goal. This goal vector could be associated with all elements of the preceding path by backward replay of place cells coding the full pathway [39, 40]. This could allow storage of an association between place cells active at the start location and the subsequent goal vector. Retrieval of the goal vector at the start location could then allow the rat to go directly to the location of food reward. If the spatial locations leading to a goal are associated with the goal vector at each goal location, and then integration is reset, a sequential series of trajectories to goals could be stored separately. The rat could then use this activity to sequentially retrieve pathways to different rewarded locations in the environment, as in some behavioral tasks [41]. The resetting of integration activity could underlie the different pattern of place cell firing shown with this type of directed task compared to open field activity. Thus, the resetting of integration could explain the shift in firing location for place cells between scavenging in an open field and following sequential trajectories between reward locations [41] as well as the shift in firing location for grid cells between open field scavenging and running on a long hairpin track [42] in which the view of each new segment could cause phase reset.

Graded persistent spiking could also hold initial head direction *θ*_HD_(0) and update this by integrating input from neurons coding angular head velocity ${\overset{˙}{\theta }}_{\text{AHV}}(t)$ in areas such as the postsubiculum [27].

## 5. PERSISTENT SPIKING COULD UNDERLIE GRID CELL FIRING

Because the neurons that show persistent firing can integrate the synaptic input from speed modulated head direction cells, and thereby can code spatial location, these persistent firing neurons could potentially be the grid cells recorded in awake behaving animals [24, 43, 44]. This section describes two potential mechanisms for persistent firing neurons to contribute to the activity of grid cells. The first mechanism involves the cyclical persistent firing shown in layer II (see Figure 1(a)). The second mechanism involves the graded persistent firing shown in layer V (see Figure 2(a)).

In a general manner, the experimental data on firing of single grid cells can be described by

(4) $$g(t)\;=\;\underset{\theta }{\prod }\cos(\omega H\overset{⇀}{x}(t)\;+\;\varphi ),$$

where *g*(*t*) is the probability of firing of the grid cell over time. The product sign Π represents multiplication of the output from each row (each head direction **θ**) of the head direction transform matrix *H* described above. The description of the experimental data here directly uses the vector representation of location over time *x*(*t*). Orientation of the grid is determined by the head directions **θ** in *H*, the spatial phase is determined by *φ*, and the spacing between fields is determined by the angular frequency *ω*. This equation resembles other representations of grid cells [18, 45] but simplifies the representation by using the head direction transform matrix.

### 5.1. Cyclical persistent firing

The pattern of periodic spatial firing of grid cells could arise from the pattern of cyclical persistent firing as shown in Figure 1(a). The tendency for persistent firing to turn on and off could contribute to grid cell firing if the oscillation could be gated by integration of input from different populations of head direction cells with different preferred angles. Simulations shown in Figures 1(b) and 1(c) show that the following equations can generate grid cell firing properties:

(5) $$\begin{array}{ll} \frac{\mathrm{d}h^{+}}{dt} & \;=\;-\omega^{2}V\left(t\right)(H{\overset{⇀}{v}}^{+}\left(t\right){)}^{3/2},\\ \frac{\mathrm{d}h^{-}}{dt} & \;=\;\omega^{2}V\left(t\right)(H{\overset{⇀}{v}}^{-}\left(t\right){)}^{3/2},\\ \frac{\mathrm{d}V^{}}{dt} & \;=\;h^{+}(H{\overset{⇀}{v}}^{+}\left(t\right){)}^{1/2}-h^{-}(H{\overset{⇀}{v}}^{-}\left(t\right){)}^{1/2},\\ g\left(t\right) & \;=\;[\prod V\left(t\right)], \end{array}$$

where *h* ^+^ represents changes in current due to positive components of the head direction input (note that this uses cosine modulated head direction input), and *h* ^−^ represents current due to negative components of the cosine modulated head direction input. Note that the equations separately use positive and negative elements of the speed modulated head direction matrix *H* transforming the rat movement velocity *v*(*t*). The parameter *ω* determines the time scaling of input effects on activity levels. In the equations, *V*(*t*) represents the voltage change in individual compartments each of which receive input from the positive and negative components of one head direction input. As noted above, the cosine modulated head direction input could be provided by summing over head direction cells with different angles of preference. The negative influence of head direction in the equation could be due to feedforward inhibition or inhibitory GABAergic projections from the postsubiculum to the medial entorhinal cortex. The function *g*(*t*) represents the firing of grid cells over time. The square brackets [] indicate that spiking occurs whenever *V*(*t*) crosses a threshold.

This pattern of activity could be obtained if neurons respond to different head direction inputs with cyclical persistent firing (Klink and Alonso, 1997), as shown in Figure 1(a). In this case, when going one direction, head direction input will cause phasic changes in firing in that direction, possibly due to build up first of calcium and then of calcium inactivation. When going the exact opposite direction, head direction input would have to activate the reverse processes, possibly reducing calcium inactivation and then reducing calcium.

Examples of grid field plots obtained with this model are shown in Figures 1(b) and 1(c). The grid fields are more stable in the trajectory data from the Moser laboratory than in randomly created trajectories (see Figure 1(b)) or in a trajectory obtained in our own laboratory (see Figure 1(c)). This indicates that the statistics of rat movement can determine appearance of the grid in this new model, and this could underlie variability in detection of grid cell firing properties depending on the trajectory of rat movement in the behavioral foraging task.

### 5.2. Graded persistent firing

As an alternative to cyclical persistent firing, graded persistent firing as shown in Figure 2(a) could provide the basis for grid cell firing. In this framework, different graded persistent firing cells start out with the same baseline frequency of spiking and provide convergent input to a grid cell that fires whenever the inputs are in synchrony. Speed modulated head direction input to different graded persistent firing cells will transiently alter the frequency and thereby the phase of firing. Therefore, if the rat moves, it shifts the frequency of a graded cell out of phase with the other cells and thereby reduces or stops the grid cell firing until the phase is shifted enough to come into phase with the other neurons. A grid cell simulated with this model is shown in Figure 2(b). This mechanism uses graded persistent firing in a manner similar to the mechanism of membrane potential oscillations described in the following section.

Both of these models will yield a pattern of firing similar to grid cells as long as the head direction cells providing input have preference angles at multiples of 60 degrees. For path integration, the head direction angles used for integration might be determined at the start location. For example, a single pyramidal cell showing persistent firing might receive input from three head direction cells that code the heading angle at the start, as well as the angle of eye direction. Rats have binocular overlap of about 60 degrees [46]. If the total visual field of one eye is 180 degrees and the center of view is at 90 degrees, then the center of view for each eye should be offset about 60 degrees from head direction. Therefore, a rat may choose angles of 0, −60, and 60 degrees for path integration. These angles have the 60-degree difference necessary for the head direction input to cause hexagonal arrays in the grid cell model. The rat can use these initial angles of view as reference angles, but if it turns far enough from the initial heading (e.g., 180 degrees from the initial head direction), then it may need to select additional reference angles at 60-degree intervals from the previous reference angles.

Some grid cells respond selectively only for certain head direction [25]. These head direction sensitive grid cells might result from the input only being suprathreshold for a population of head direction cells responding near one preferred angle, with input being subthreshold from other populations of head direction cells coding other preferred angles (e.g., at 60 or 120 degrees differences).

## 6. INTEGRATION BY MEMBRANE POTENTIAL OSCILLATION PHASE CAN CODE LOCATION

As an alternative mechanism for path integration, the phase of membrane potential oscillations in medial entorhinal stellate cells can also be used to integrate speed modulated head direction input. This mechanism was proposed in a model of grid cells developed by Burgess et al. [18] and O'Keefe and Burgess [47]. This mechanism uses the physiological fact that depolarizing inputs to stellate cells cause a change in frequency of membrane potential oscillations [16]. This could change oscillation phase based on an integral of the depolarizing input.

### 6.1. Model of grid cells using membrane potential oscillations

This computational model shows how activity of a single grid cell could arise from membrane potential oscillations within that cell modulated by depolarizing input from head direction cells:

(6) $$g(t)\;=\;[\prod \left(\cos\,\;\omega t\;+\;\cos(\omega t\;+\;\omega \beta_{H}\int_{0}^{t}\;H(\overset{⇀}{v}(\tau )d\tau \;+\;x_{0}))\right)],$$

where *g*(*t*) is the firing in time and space of a single modeled grid cell. *ω* represents the baseline angular frequency of membrane potential oscillations (2∗*π*∗*f*) in different portions of the neuron. **β*_H_* is the experimentally determined scaling factor relating membrane potential oscillations to grid cell spacing. The input from head direction cells is determined by the matrix *H* and the velocity vector $\vec{v}$. The inner product of each row of *H* with the velocity vector $\vec{v}$ represents input to one dendrite from one head direction cell modulated by the speed of the rat. This input alters the frequency of dendritic membrane potential oscillations and thereby shifts the phase of the dendritic oscillations in proportion to the integral of the velocity vector over time. Both the starting location of the rat and the spatial phase of the grid cell are combined in the initial location vector *x*_0_. This initial location vector is also transformed by the matrix *H*. The square brackets [] represent a Heaviside step function generating a spiking output for each time point when the product crosses a threshold (set at 1.8).

This model generates grid cell firing fields with spacing between fields dependent upon the frequency of membrane potential oscillations [16–18]. A grid cell created with this model is shown in Figure 2(c). The model generated the prediction that the systematic change in spacing of grid cell firing fields along the dorsal to ventral axis of entorhinal cortex would depend upon a systematic difference in frequency of membrane potential oscillations in entorhinal neurons. This prediction was tested and supported by whole cell patch recordings from entorhinal layer II stellate cells [16]. Based on experimental data alone, it appears that membrane potential oscillation frequency *f* is scaled to grid cell spacing *G* by a constant factor *f* ∗ *G* = *H* [16, 17].

As noted above, membrane potential oscillations appear in specific medial entorhinal populations such as layer II stellate cells and layer V pyramidal cells, but not in other cells such as layer II pyramidal cells, or neurons in medial entorhinal layer III, or in the lateral entorhinal cortex. Based on these data, the generation of grid cells responses based on membrane potential oscillations would only occur in layer II stellate cells and layer V pyramidal cells, and would appear in other neurons due to network interactions or due to the persistent spiking mechanisms described above.

### 6.2. Grid cell activity codes location

In the model, the depolarizing input from head direction cells increases the frequency of membrane potential oscillations in proportion to the velocity vector. This shift in frequency alters the phase of oscillations in proportion to the integral of the velocity vector transformed by head direction: $\int_{t=0}^{T}H(\overset{⇀}{v}(t)dt\;+\;x_{0})\;=\;H\overset{⇀}{x}(T)$. In addition, the interference pattern increases and decreases in proportion to the difference in oscillation frequency of the soma and dendrite, so that the vector of angular phases of interference is $\varphi (t)\;=\;\omega \beta H\overset{⇀}{x}(t)$ (see Figures 3(b) and 3(c)) and the equation for grid cell activity can be written for location as $g(t)\;=\;[\prod \cos(\omega \beta_{H}H\vec{x}(t))].$

The location can be extracted from grid cell phase by using the inverse of the head direction transform matrix as follows: $\overset{⇀}{x}(t)\;=\;H^{-1}\overset{⇀}{\varphi }(t)/\omega \beta$. Figure 3(d) shows the trajectory obtained from this inverse transform of phase.

### 6.3. Theta phase precession

In addition to replicating the spatial periodicity of grid cell firing fields, the model based on interference of membrane potential oscillations also replicates experimental data showing systematic changes in phase of grid cell firing relative to network theta rhythm oscillations [48], a phenomenon known as theta phase precession. The phenomenon of theta phase precession was initially shown for place cell firing in the hippocampal formation [49, 50] and was proposed to arise from the interaction of network theta rhythm oscillations with cellular theta rhythm oscillations [49, 51, 52]. As an alternative model, precession was proposed to arise from the readout of sequences of place cell activity [53–55].

The oscillatory interference model of grid cells [18, 47] extended the models of hippocampal phase precession and can account for grid cell phase precession [48]. In the oscillatory interference model, the interference has two components that appear physiologically: (1) as described above, the size of grid fields is determined by the envelope that has a frequency depending on the difference of the two angular frequencies *ω*_*d*_ − *ω*_*s*_, and (2) the pattern of phase precession depends on a higher frequency component that corresponds to the mean of the two frequencies (*ω*_*s*_ + *ω*_*d*_)/2. The phase of this second-high frequency component of the summed oscillation is $\varphi_{\text{sum}}\;=\;\omega t\;+\;\omega \beta \int_{0}^{t}\;H\overset{⇀}{v}(\tau )d\tau /2$. The spiking will occur near the peak phase of this summed oscillation which is equal to some multiple *n* of the full cycle: *φ*_sum_ = *n*2*π*.

The spiking caused by the phase of the summed oscillations can then be plotted relative to the network theta rhythm by plotting the phase of the soma at the time of each spike. This can be obtained analytically from the above equation if we consider the case of the movement at a constant speed continuously in the preferred direction of one head direction cell. In this case, the integral of head direction for that cell is simply the integral of speed, which is equal to the location $x\;=\;\int_{0}^{t}H\overset{⇀}{v}(\tau )d\tau$. Note also that the phase of the soma oscillations is the product of soma frequency and time: *φ*_soma_ = 2*π* *f* *t*. Therefore, the equation for the phase of the summed oscillation can be reduced to *φ*_sum_ = *φ*_soma_ + *ω* *B* *x*/2.

**Figure 4:**
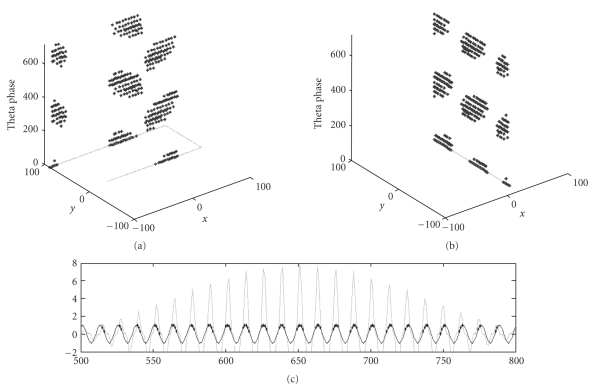
Simulation of theta phase precession in grid cell model based on membrane potential oscillations. (a) Theta phase plotted on vertical axis as a simulated rat runs through a grid cell firing field in west to east and east to west directions. (b) Theta phase during run from south to north. (c) Spike times (filled circles) of summed oscillations in a neuron relative to oscillation in the soma of that neuron (negative of network theta oscillation).

Plotting of theta phase precession essentially involves plotting the timing of spikes (which occur when *φ*_sum_ = *n*2*π*) relative to the phase of the network oscillations (which here correspond to the phase of the soma because the soma is being driven by network oscillations with fixed frequency *ω*). Thus, the vertical axis of a plot of theta phase precession shows the phase of the soma oscillation at the time of each spike: *φ*_soma_ = *n*2*π* − *π* *f* *B* *x* plotted relative to location *x* on the horizontal axis. Figure 4 shows the plotting of spikes in the simulation during runs on a linear track through the firing field of the neuron. Note that the phase precession in this model resembles the phase precession found in experimental data [50] but only covers about 180 degrees of the network theta oscillation cycle.

**Figure 5:**
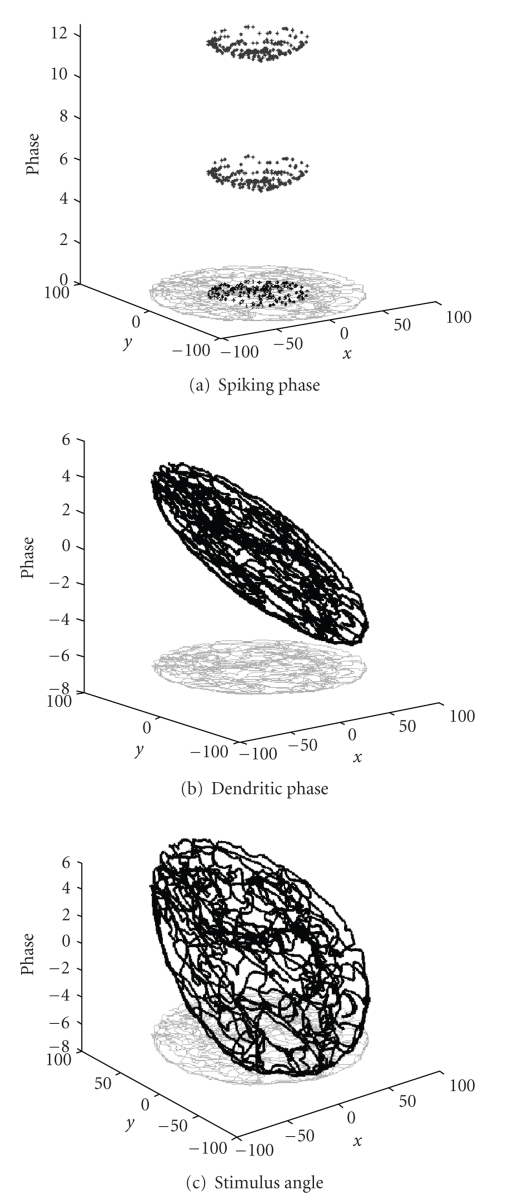
Phase relative to location in the environment. (a) Spiking phase due to precession (with refractory period). Note that phase depends upon location, but is circularly symmetric. (b) Dendritic phase of oscillations contains more complete continuous representation of location. (c) Plot of the angle of a single distal visual stimulus as a rat moves around in an environment, indicating similarity of allocentric stimulus angle to integrated dendritic phase in a grid cell.

Note that the phase precession for a single direction gives a partial readout of the phase code of location, but when considering the phase in two-dimensional space, it confounds the phase of the two or more dendrites, so it is radially symmetric and dependent upon the direction of trajectory through the field (see Figure 5(a)). Thus, the phase precession code is less accurate for use in path integration, in contrast to the overall mean firing rate that would be observed in a grid cell due to persistent firing with a very large firing field, which could code location for distances smaller than one half the spacing between two grid fields (e.g., for 80 cm spacing, distances less than 40 cm could be coded).

### 6.4. Mechanism for path integration

As noted above, path integration involves an animal using its self-motion cues to maintain an accurate representation of angle and distance from start position. In the grid cell model, the update of oscillation phase by speed-modulated head direction integrates the velocity vector, thereby integrating the distance from the starting position in specific directions determined by *H*. If oscillation phases are reset to zero at the starting location, the return vector **r** giving distance and direction back to the starting location can be obtained by applying the inverse of the head direction matrix to the dendritic phase vector at any position $\overset{⇀}{r}=-H^{-1}\overset{⇀}{\varphi }/\omega \beta$. Figure 3(a) shows that after resetting phase at the starting location, applying the inverse head direction transform to the dendritic phase vector, and taking the negative of this vector gives the direction and angle directly back to the starting location (dotted line).

### 6.5. Interaction of path integration and visual stimuli

Path integration based on idiothetic cues alone builds up substantial error [34] that is usually corrected by comparison with sensory cues such as visual stimuli [35]. Grid cells show dependence upon visual stimuli in the environment. They maintain the same properties when returned to a familiar room [24], and they change their scale during a period of time after manipulations of environment size [44]. This influence of visual stimuli could result from the fact that the angle to visual stimuli has the same properties as phase of grid cell oscillations. Figure 5 shows how the angle of a single distal visual stimulus changes as a rat moves in a manner that is consistent with the change in phase of individual dendrites of a single modeled grid cell.

Alternately, the grid cell could be influenced by the angle and distance to visual stimuli, and neural mechanisms could update the expected angle and distance to visual stimuli in a manner similar to the mechanism for updating the angle and distance from start location (return vector). This requires updating the angles of the initially selected stimuli (that might be determined by eye direction) according to the direction and velocity of movement. This will update the expected absolute angle of visual stimuli. The further computation of expected relative angle (the actual visual input) requires combining absolute angle with current head direction. The process of updating head direction could depend upon input from cells coding angular velocity of movement. These have been shown in the postsubiculum [27, 56] as well as in structures including the anterior dorsal thalamic nucleus [29]. Grid cells appear to be more consistent when there are clear barriers on the edge of the open field, suggesting that rats might use the vertical angle of a boundary to judge distance.

The basic grid cell model assumes speed modulation of head direction cells, but most head direction cells show stable persistent firing even when the rat is motionless. In contrast, place cells show more speed modulation. In keeping with the physiological data that shows stable persistent firing for head direction cells and speed dependent firing for place cells, it might be appropriate to represent state as the static head direction cell activity combined with visual stimulus angle, and to use the speed-dependent activity of place cells as the action of the rat.

### 6.6. Grid cell phase represents continuum of locations for reinforcement learning

The grid cells can be used as a representation of state for goal directed behavior. Many reinforcement learning theory models have used discrete representations of state for goal directed behavior. However, this causes difficulties for representing movement in continuous space. The phase of oscillations in the grid cell models is a continuous representation of space that can be updated in a continuous manner by actions held by persistent spiking. As noted previously [16, 57], this could allow grid cells to provide an effective mechanism for representations of state and action in continuous space and time.

## 7. MODEL OF EPISODIC MEMORY

**Figure 6:**
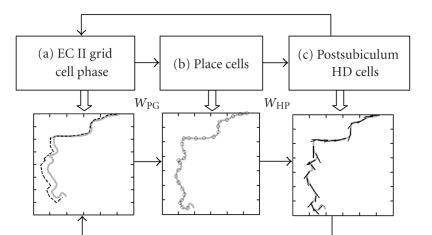
Model of episodic encoding and retrieval of trajectories. Top: Schematic representation of connectivity between grid cells, place cells, and head direction (HD) cells. Bottom: example of trajectory retrieval activity in each region. Trajectory experienced during encoding is shown in gray. (a) The location coded by the oscillation phase of entorhinal grid cell membrane potential is plotted as a dashed line that follows the actual trajectory in gray. Grid cell phase is put through the inverse head direction transformation to obtain coded location. Phase shifts are driven by retrieved head direction until next place cell is activated, then phase moves in new direction dependent on next active head direction cell. (b) Sequentially activated place cell representations are shown as open circles. (c) Each place cell activates a corresponding head direction representation, with direction shown as a short, straight, black line. This drives the next period of update of the grid cell phase.

The interaction of head direction cells and grid cells described here provides a potential mechanism for episodic memory involving the storage of trajectories through space and time [57]. As shown in Figure 6, this model uses a functional loop that encodes and retrieves trajectories via three stages: (1) head direction cells *h*(*t*) update grid cells, (2) grid cells *g*(*t*) update place cells, and (3) place cells *p*(*t*) activate associated head direction activity [57]. This model is consistent with the anatomical connectivity (see Figure 6). The head direction cells could update grid cells via projections from the postsubiculum (dorsal presubiculum) to the medial entorhinal cortex [58–60], causing updating of persistent firing as described above, or influencing the phase of membrane potential oscillations [16–18]. Grid cells can update place cells via the extensive projections from entorhinal cortex layer II to dentate gyrus and CA3 and from layer III to region CA1 [45, 61]. The connectivity from grid cells to place cells could be formed by different computational mechanisms [45, 62, 63]. Place cells can become associated with head direction activity via direct projections from region CA1 to the postsubiculum [58], or via indirect projections from region CA1 to the subiculum [61, 64], and projections from the dorsal and distal regions of the subiculum to the postsubiculum and medial entorhinal cortex [65], both of which contain head direction cells.

During initial encoding of a trajectory in the model, the head direction cell activity vector would be set by the actual head direction of the rat during exploration, and associations would be encoded between place cell activity and head direction activity. These associations would be stored in the form of a synaptic connectivity matrix *W*_HP_ with strengthened connections between active place cells *p*(*t*) and the head direction cell activity vector *h*(*t*) as follows:

(7) $$W_{\text{HP}}\;=\;{\underset{p}{\sum }{\overset{⇀}{h}}_{p}(t)\overset{⇀}{p}(t{)}^{T}}^{}.$$

In this equation, head direction vectors associated with individual place cell locations are identified with the place cell index *p*. During retrieval, the head direction activity depends upon synaptic input from place cell representations as follows: *h*(*t*) = *W*_HP_ *p*(*t*).

This model has the capacity for performing episodic encoding and retrieval of trajectories in simulations [57], including trajectories based on experimental data or trajectories created by an algorithm replicating foraging movements of a rat in an open field [17]. During encoding, a series of place cells *p*(*t*) is created associated with particular locations *x*_*p*_ = *x*(*t*). Each place cell is also associated with input from the grid cell population activity *g*(*t*) and with the head direction vector *h*_*p*_ = *h*(*t*) that occurred during the initial movement from that location. For retrieval, the simulation is cued with the grid cell phase vector *φ*(*t*_0_) and head direction vector *h*(*t*_0_) present at the start location. The head direction vector updates the grid cell phase vector *φ*_*d*_(*t*), which alters the activity of grid cells. The grid cell firing drives place cells *p* associated with subsequent locations on the trajectory. These mechanisms are summarized in Figure 6.

The activation of each new place cell activates a new head direction vector *h*_*p*_ associated with that place cell. This new head direction vector then drives the further update of dendritic phases of grid cells. This maintenance of the head direction vector might require graded persistent spiking [8] of head direction cells in deep layers of entorhinal cortex. Essentially, the retrieval of the place cell activity representing the state drives the retrieval of the new head direction vector representing the action from that state. This action is then used for a period of time to update the grid cell state representation until a new place cell representation is activated.

Because retrieval of the trajectory depends on updating of phase by head direction cells, this allows retrieval of a trajectory at a time course similar to the initial encoding. This can allow effective simulation of the slow time course of place cell replay observed in previous experimental data collected during REM sleep [66]. The spread of activity from place cells to cells coding head direction could contribute to patterns of firing in the postsubiculum that appear as cells responding dependent on both place and head direction [31]. These cells might code the action value for retrieval of a trajectory from a particular location, firing only when actual head direction matches the head direction previously associated with specific place cell activity. The strong theta phase specificity of these cells could be due to separate dynamics for encoding and retrieval within each cycle of theta rhythm [67]. These cells might selectively fire during the retrieval phase.

### 7.1. Enhancement by arc length cells

The retrieval mechanism mediated by place cells can be enhanced by inclusion of cells that fire dependent upon the arc length of the trajectory [57], or by the time interval alone [17]. These types of responses help prevent a breakdown in trajectory retrieval caused by overlaps in the trajectory. The associations between place cells and head direction cells cannot disambiguate between two segments of a trajectory passing through the same location with different head directions. However, coding of arc length or time since the start of the trajectory can disambiguate these overlapping locations. Oscillatory interference between neurons directly modulated by speed but not head direction can activate arc length cells coding arc length from the start of a trajectory, or from the last time that oscillations are reset along the trajectory. Simulations based on this coding of arc length can account for many features of unit recording data in behavioral tasks. Persistent spiking in layer III of entorhinal cortex could provide a means for driving the coding of arc length (or time) along a trajectory. Persistent spiking in layer III with specific frequencies [6] could activate neurons in region CA1 with different phases relative to arc length (or time) on a trajectory. During retrieval, arc length cells from one portion of a trajectory can activate associated speed modulated cells to trigger the next arc length cell along a trajectory. This retrieval process can be accelerated or decelerated via modulation of the frequency of entorhinal oscillations during persistent firing.

### 7.2. Predictions of arc length model

Since the output of arc length cells is essentially dependent upon distance, a simple manipulation of running distance should directly influence spatial specificity of arc length cells. For example, in a rat running continuously clockwise around a circular track with a circumference of two meters, an arc length cell may match the periodicity of the track and display a stable place field somewhere on the track. Here, the cell is firing at an arc length of two meters. If we expand the track by a small amount to say, a circumference of 2.1 meters, the arc length cell would be expected to continue to fire at two-meter intervals, thus the field will translocate in the counterclockwise direction (or backwards in relation to the rat) by 10 centimeters for each lap.

The reset version of the arc length coding model assumes that oscillations reset at a specified location or during a key event such as food reward. This is supported experimentally given that the theta oscillation does reduce when an animal stops or consumes food. By using the same manipulation on the circular track as before, a similar but quite different prediction surfaces. Here, the field will shift counterclockwise abruptly, but will remain at that location for subsequent laps. This location stability is a direct consequence of stability of the food reward location since now the oscillatory interference is anchored to the food reward, and not from the previous location that the cell had discharged. Interestingly, the distance a field moves will be linearly proportional to the distance the original field was from the food reward location. Thus, a field will move 10 centimeters only if the field was originally located at the end of a lap (at the feeder), and a field will shift 5 centimeters if the original field was located at the midpoint of the lap.

The reset model prediction of the expanded circular track leads us to a further prediction. Since the discharge of an arc length cell in the reset model is dependent on and anchored to the reward location, a manipulation of the location of food reward will cause a relative movement of the location of the arc length's discharge. For example, the movement of the food reward by 10 centimeters in the clockwise directly will cause an arc length cell to correspondingly shift its field 10 centimeters in the clockwise direction.

## 8. CONCLUSIONS

The cellular physiological phenomena described in this paper provide mechanisms important for behavioral functions including path integration and the episodic encoding and retrieval of trajectories. Detailed computational models demonstrate the potential behavioral role of cellular mechanisms of persistent spiking and membrane potential oscillation, demonstrate how these could underlie unit recording data such as grid cell firing, and generate predictions for future experimental studies.
